# *Lactobacillus casei*’s Antitumor Potential in Colorectal Cancer: Exploring Mechanisms—A Systematic Review

**DOI:** 10.1155/bmri/4216722

**Published:** 2025-11-09

**Authors:** Mahdi Abdorrashidi, Mohammad Heiat, Amin Vesal Yeganeh, Amirmohammad Tohidinia, Arman Alizadeh, Ali Ramazani, Hamed Gholizadeh, Toktam Pouraskar, Mohammad Hossein Peypar

**Affiliations:** ^1^ Student Research Committee, Baqiyatallah University of Medical Sciences, Tehran, Iran, bmsu.ac.ir; ^2^ Baqiyatallah Research Center for Gastroenterology and Liver Diseases (BRCGL), Clinical Sciences Institute, Baqiyatallah University of Medical Sciences, Tehran, Iran, bmsu.ac.ir; ^3^ Research Center for Obesity and Metabolic Diseases, Clinical Sciences Institute, Baqiyatallah University of Medical Sciences, Tehran, Iran, bmsu.ac.ir; ^4^ Student Research Committee, Dezful University of Medical Sciences, Dezful, Iran, dums.ac.ir

**Keywords:** apoptosis, colorectal cancer, immunomodulation, *Lactobacillus casei*, short-chain fatty acids

## Abstract

Colorectal cancer (CRC) is the third most common cancer worldwide. Around 1.8 million people were diagnosed with CRC in 2018, and 881,000 died. The limitations of chemotherapy and radiotherapy, as well as the uncertainty of CRC‐specific therapies, encourage the development of alternative CRC prevention, treatment, and control measures. Probiotics are being studied as a strategy for preventing and treating CRC due to their potential health benefits. *Lactobacillus casei* (*L. casei*) shows promise in reducing tumor growth and cancer cell survival in CRC, according to recent studies. Due to the varying efficiency of probiotics depending on the specific strain, substantial research has been conducted on the *L. casei* strains to explore their potential anticancer effects in CRC. In this study, we aimed to conduct a systematic review of exploring the various mechanisms of *L. casei* strains to facilitate the development of effective probiotic supplements to complement standard CRC therapy. We conducted a meticulous search on Scopus, PubMed, Embase, and Web of Science. Initial research resulted in 433 records, from which 412 papers were excluded by reason. The remaining 21 papers were categorized into four topics. These papers discuss several mechanisms involved in anticancer properties against CRC, including apoptosis induction and antiproliferation activity, immunomodulation, gut microbiome, intestinal barrier function modulation, and detoxifying carcinogens. Our findings suggest that using the potential strains of *L. casei* in combination therapy and targeted therapy, along with conventional drugs, could be a promising approach against CRC.

## 1. Introduction

Colorectal cancer (CRC) is among the most frequent cancers worldwide. In 2018, around 1.8 million new cases of CRC were diagnosed, resulting in approximately 881,000 mortalities [1].

CRC incidence and mortality rates exhibit considerable global variation. CRC ranks as the third most prevalent cancer in men and the second most prevalent cancer in women worldwide. The incidence of CRC is notably higher in males compared to women [1–3].

CRC mortality in the United States declined steadily from 1999 to 2020, largely driven by widespread adoption of colonoscopy screening, early removal of polyps, and advances in multimodal treatments, including surgery, chemotherapy, radiation, and immunotherapy. Despite this progress, African Americans continue to experience the highest mortality rates, while younger White populations show an alarming rise in early‐onset CRC deaths [4]. Furthermore, the mortality rate is still increasing in many countries with limited healthcare resources and infrastructure [5].

Several environmental and lifestyle clinical variables are linked to a slight or uncertain rise in the risk of CRC. Although observational studies have consistently shown these relationships, the causal relationship between these factors has not been definitively demonstrated. The variables contributing to this include obesity, Type 2 diabetes, insulin resistance, intake of processed meat, tobacco usage, alcohol consumption, and the use of deprivation therapy and cholecystectomy [6–10].

Various bacterial and viral factors, such as *Streptococcus bovis*, *Helicobacter pylori*, JC virus, human papillomavirus, *Fusobacterium nucleatum*, disease‐causing strains of *E. coli* that produce colibactin (a toxic substance that damages DNA), and a decrease in the variety of bacteria in the gut microbiome, have been proposed as factors that increase the risk of CRC. Emerging evidence confirms that the gut microbiome may play a significant role in determining the connection between diet and CRC [11–16].

Among the various microorganisms, *Lactobacillus casei* (*L. casei*), a member of the lactic acid bacterial (LAB) group, is particularly noteworthy [17]. The consumption of LAB has been found to be effective in treating multiple disorders, including intestinal inflammation, diarrhea, allergic diseases, and psychiatric problems [18–20]. Research indicates that some of them possess immunomodulatory characteristics and can effectively enhance the host’s defense mechanisms, particularly in relation to colon cancer.

Several strategies have been suggested for probiotics, such as altering the composition of the gut microbiota, inhibiting pathogenic species, enhancing the protective barrier of the intestinal lining, regulating immunological responses, and promoting anticancer and proapoptotic effects [21–23].

Besides this, bioactive compounds produced by these microorganisms, such as bacteriocins, short‐chain fatty acids, and diverse polysaccharides, have also been identified as key mediators in these processes, emphasizing the multifaceted therapeutic potential of probiotics in CRC prevention [24].

The *L. casei* group comprises *L. casei*, *Lactobacillus paracasei*, and *Lactobacillus rhamnosus*, which are closely related and have been the subject of extensive research due to their health‐promoting properties. They are recognized as safe by the US Food and Drug Administration (FDA) and European Food Safety Authority (EFSA) [25].

The *L. casei* strains are used in the food industry and have potential therapeutic applications for diseases associated with gut microbiota disturbances. They demonstrate promise in prophylactic or therapeutic use for various conditions, including allergies, obesity, and cancer, due to their ability to maintain a healthy gut microbiota and modulate the immune system [25, 26]. Recent studies have indicated that *L. casei* may play a role in reducing tumor volume and inhibiting cancer cell viability, suggesting a promising candidate for CRC treatment [27].

Indeed, conventional treatments for CRC, such as surgery, radiation therapy, and chemotherapy, have limitations. These limitations stem from the inability to distinguish between normal and malignant cells, as well as the risk of inducing cancer in surrounding tissues due to radiation exposure [28, 29]. Nevertheless, despite probiotic therapy holding a considerable promise over traditional treatments, it is important to note that the effectiveness of probiotics is highly dependent on the type of strains, and results from one organism cannot be generalized to others. The various antitumor mechanisms exhibited by different *L. casei* strains on various types of CRC cells highlight the need to understand these mechanisms [30].

This understanding can guide the development of efficient bio supplements to complement standard CRC therapy. Therefore, in this systematic review, we aim to investigate the possible mechanisms involved in *L. casei*’s antitumor effects against CRC.

## 2. Method

The investigation was carried out in accordance with the PRISMA 2020 guideline [31]. Two researchers independently searched through databases, and a third researcher handled any inconsistencies.

### 2.1. Data Source and Search Strategy

We meticulously searched Scopus, PubMed, Embase, and Web of Science up to December 2023 to find literature on how *L. casei* can act as an antitumor agent in CRC. The search method employed in our study includes the following keywords: “colorectal”, “tumor”, “Carcinom ^∗^”, “Neoplasm”, “cancer”, “malignancy”, “colorectal neoplasia”, “colorectal neoplasm”, “colorectal tumor”, “colorectal tumorigenesis”, “neoplastic colorectal”, “colorectal cancer”, “colon”, “rectal”, “colorectal malignancy”, “colorectal malignancies”, “recto colonic cancer”, “*lactobacillus*”, “*casei*”, “*Lactobacillus casei*”, “*bacillus casei*”, “*lactobacilli casei*”, and “*Lacticaseibacillus casei*.”

### 2.2. Inclusion and Exclusion Criteria

The inclusion criteria comprise experimental studies that investigated the mechanism of action of *L. casei* strains against CRC. The exclusion criteria for our studies included studies in languages other than English, review studies, letters to the editor, case studies, and failure to evaluate the mechanisms of *L. casei*.

### 2.3. Data Extraction

The extracted items from the articles include the authors’ names, year of publication, study setting, bacterial strain, *L. casei* dosage, and the obtained results. Additionally, information on the mechanisms of *L. casei* on tumor tissue and the types of cell lines or animals used was extracted.

### 2.4. Quality Assessment

The risk of bias in vivo studies was assessed using SYRCLE’s risk of bias tool [32]. To facilitate comprehensive interpretation, we used a visualization tool for risk of bias assessments related to a systematic review (the robvis tool). This tool provides a visualization of findings using a generic dataset design [33].

On the other hand, the quality assessment of in vitro studies was evaluated by the authors using a custom‐built tool for assessing the quality of the studies. This tool was developed in accordance with research conducted by Golbach et al. [34] and a systematic review [35]. Two authors, with the final decision made by M.H., customized the tool to include the following domains: (i) cell origin and cell type, (ii) bacterial strain, (iii) duration of exposure, (iv) dosage of bacteria, (v) control group, (vi) methodology for control and exposure treatment, (vii) clear descriptions of the methods used for each analysis, (viii) temperature control, and (ix) statistical measures for assessing the quality of in vitro studies.

## 3. Results

### 3.1. Search Results and Characteristics

**Figure 1:**
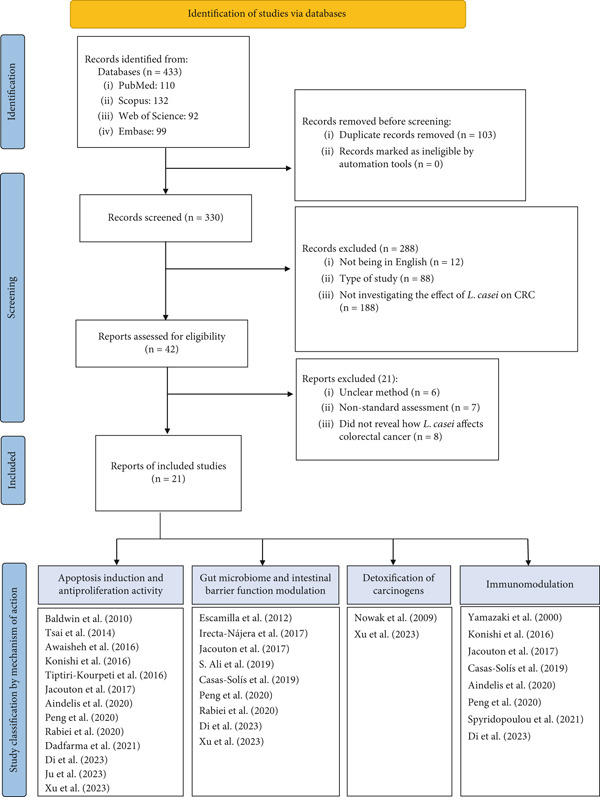
PRISMA flowchart for selection and classification of relevant studies.

The initial database search yielded 433 records. After removing 103 duplicates, title and abstract review led to the exclusion of 288 papers, including review studies, letters to the editor, case reports, and non‐English articles. Full‐text assessment of the remaining 42 articles resulted in the exclusion of 21 more due to methodological ambiguities, nonstandard assessments, or a failure to evaluate the mechanisms of *L. casei*. Consequently, 21 articles met the final inclusion criteria (Figure 1).

### 3.2. Quality Assessment

**Figure 2:**
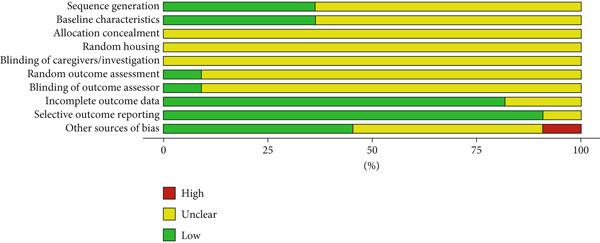
Measurement of the risk of bias assessment for in vivo studies using SYRCLE’s risk of bias tool. The studies reveal that insufficient reporting on several methodological aspects, including randomization, blinding, and housing practices, prevents a definitive judgment of low risk of bias for many items in SYRCLE’s RoB tool.

**Figure 3:**
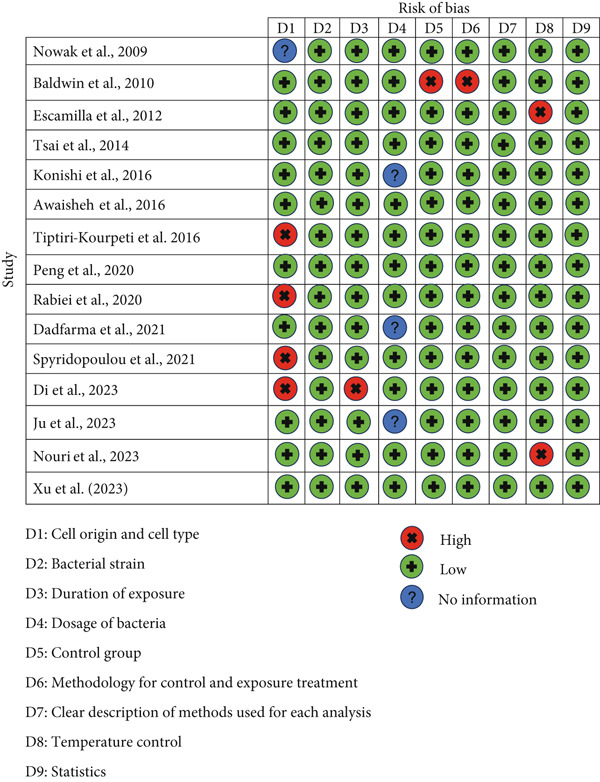
The risk of bias in in vitro studies was assessed by the authors using a custom‐built tool to evaluate the study quality.

The animal studies reveal that insufficient reporting on several methodological aspects, including randomization, blinding, and housing practices, prevents a definitive judgment of low risk of bias for many items in SYRCLE’s RoB tool. However, most in vitro studies were judged as low risk across almost all domains (Figures 2 and 3).

### 3.3. *L. casei* Exhibits Anti‐CRC Mechanisms In Vitro and In Vivo

**Figure 4:**
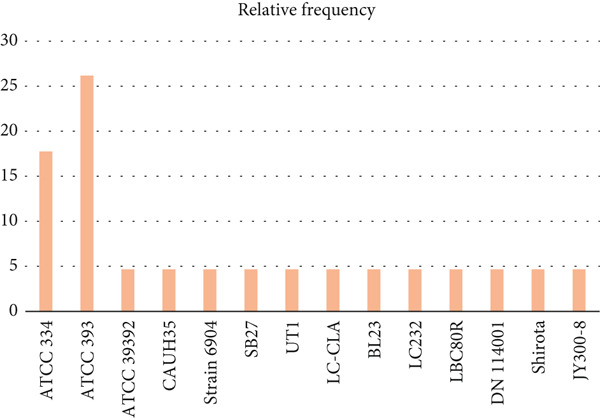
Several strains of *L. casei* were employed in the selected studies. The *L. casei* ATCC 393 and *L. casei* ATCC 334 strains appear to be the most often used in studies.

**Figure 5:**
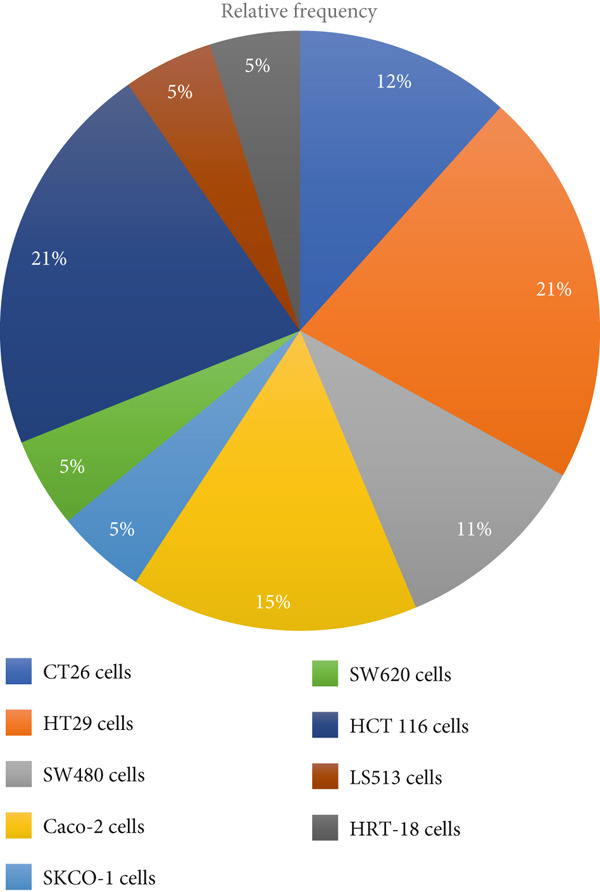
Several types of CRC cell lines were utilized in the studies. CT26 and HT29 cells are the most commonly used.

After conducting a systematic review on the potential of *L. casei* in managing CRC, we determined that the studies utilize a variety of in vitro and in vivo models to investigate the mechanisms through which *L. casei* exerts its anticancer effects (Table 1). Figures 4 and 5 depict the different strains of *L. casei* and CRC cell lines that were employed in the selected studies.

**Table 1:** Data extracted from the selected articles.

**Author (year)**	**Study setting/animal or cell line**	***L. casei* strain used**	**Dosage**	**Key results**	**Ref.**
Yamazaki et al. (2000)	In vivo/Sprague–Dawley rats	Shirota	2.1 × 10^10^ cells/g	Reduced ACF, restored CD8+ lymphocytes, and lowered tumor incidence	[36]
Nowak and Libudzisz (2009)	In vitro/–	DN 114 001	10^9^ CFU/mL	Reduced genotoxicity of mutagens without affecting bacterial viability	[37]
Baldwin et al. (2010)	In vitro/LS513 cells	LBC80R	10^8^ CFU/mL	Enhanced 5‐FU‐induced apoptosis via cleaved Caspase‐3 activation and *p21* downregulation	[38]
Escamilla et al. (2012)	In vitro/HCT116 cells	ATCC 334	25% *v*/*v* cell‐free supernatants	Reduced invasion via decreased MMP‐9 and increased ZO‐1 expression	[17]
Tsai et al. (2015)	In vitro/SW480 and Caco‐2	ATCC 334	15, 30, 60, or 90 *μ*g/mL of peptide m2163 or m2386	Peptides triggered apoptosis through extrinsic and intrinsic pathways	[39]
Konishi et al. (2016)	In vitro/Caco‐2, SKCO‐1, and SW620	ATCC 334	—	Induced apoptosis via JNK and DDIT3 with stronger tumor suppression than chemotherapy	[40]
Awaisheh et al. (2016)	In vitro/Caco‐2 and HRT‐18	LC232	—	Inhibited proliferation and reduced viability of colorectal cancer cells	[41]
Tiptiri‐Kourpeti et al. (2016)	In vitro/HT29 and CT26	ATCC 393	10^8^ or 10^9^ CFU/mL	Induced apoptosis, increased TRAIL, and downregulated survivin and cyclin D1	[42]
	In vivo/BALB/c mice	ATCC 393	10^9^ CFU	Reduced tumor growth by 81.7%, enhanced TRAIL‐mediated apoptosis, and downregulated survivin	
Irecta‐Nájera et al. (2017)	In vivo/BALB/c mice	ATCC 393	10^6^ CFU	Lowered ACF and suppressed polyamine metabolism	[43]
Jacouton et al. (2017)	In vivo/C57BL/6 mice	BL23	5 × 10^9^ CFU/mL	Prevented tumor development, reduced Ki67, and increased Caspase‐7/9 expression	[44]
Casas‐Solís et al. (2019)	In vivo/BALB/c mice	ATCC 393	10^6^ CFU	Prevented proinflammatory cytokine increase and enhanced IL‐17A and Tregs	[45]
Ali et al. (2019)	In vivo/Swiss mice	ATCC 393	2 × 10^9^ CFU/0.3 mL	Decreased tumor markers, suppressed ACF, increased expression of phospho‐JNK‐1, modulated *β*‐catenin/GSK3*β*, and enriched *Akkermansia* and *Turicibacter* genera	[46]
Aindelis et al. (2020)	In vivo/BALB/c mice	ATCC 393	10^9^ CFU	Suppressed tumor growth by 60%, enhanced immune responses including IFN‐*γ* and T cells, and increased cleavage of Caspase‐3 and PARP1 in tumor extracts	[21]
Peng et al. (2020)	In vitro/HCT116 cells	ATCC 334, LC‐CLA	—	Suppressed NF‐*κ*B and downregulated COX‐2, PGE‐2, and proliferation genes	[47]
	In vivo/BALB/c mice	ATCC 334, LC‐CLA	10^9^ CFU/mL	Reduced proinflammatory cytokines, boosted IL‐10/TGF‐*β*, and modulated gut microbiota	
Rabiei et al. (2020)	In vitro/HCT116 cells	UT1	0.5, 1, 1.5, 2, and 5 × 10^7^ CFU/mL	Reduced proliferation and induced apoptosis	[48]
Dadfarma et al. (2021)	In vitro/SW480 cells	ATCC 39392	—	Promoted apoptosis in colorectal cancer cells	[49]
Spyridopoulou et al. (2021)	In vitro/HT29 and CT26	ATCC 393	10^8^ CFU or 15 *μ*g SeNps	Se‐enriched strains inhibited growth, induced apoptosis via ROS, and improved immunity	[50]
	In vivo/BALB/c mice	ATCC 393	10^9^ CFU or 150 *μ*g SeNps	SeNp‐enriched bacteria showed the strongest tumor suppression	
Di et al. (2023)	In vivo/C57BL/6 mice	SB27	25/50/100 mg/kg of SB27 polysaccharide	Suppressed colon cancer, reduced proinflammatory markers, activated Caspase‐3/8/9, restored SCFAs, and suppressed HINT2 ubiquitination	[51]
	In vitro/HCT116 cells	SB27	25/50/100 *μ*M of SB27 polysaccharide	Inhibited proliferation and migration, induced mitochondrial damage, and increased Caspase‐3/8/9 activity	
Ju et al. (2023)	In vitro/HT29 cells	CAUH35	0, 5, 25, 100, and 200 *μ*g/mL of MucBP36R or MucBP36S proteins	MucBP36R variant suppressed proliferation, while MucBP36S showed no effect	[52]
Xu et al. (2023)	In vitro/HT29, HCT116, and CT26 cells	JY300‐8	10^6^ CFU/mL	Inhibited proliferation of HT29 (72.0%) and HCT116 (66.8%) cells	[53]
	In vivo/BALB/c mice		10^9^ CFU/mL	Reduced tumor formation rate, suppressed tumor growth, and improved survival	
Haji Mehdi Nouri et al. (2024)	In vitro/HT29 cells	Strain 6904	3.125, 6.25, 12.5, 25, 50, and 100 mg/mL of the LC‐SeNPs1	Increased apoptosis and reduced proliferation via sub‐G1 arrest	[54]

Abbreviations: 5‐FU: 5‐fluorouracil; ACF: aberrant crypt foci; SCFAs: short‐chain fatty acids; SeNps: spherical nano‐Se.

#### 3.3.1. *L. casei*–Mediated Apoptosis and Antiproliferation in CRC Models

Investigations into the effects of various *L. casei* strains and their derived products have demonstrated antiproliferative effects and the induction of apoptosis in CRC models, both in vitro and in vivo. These effects are often associated with specific molecular mechanisms impacting cell cycle progression, death receptor pathways, and mitochondrial function.

a. Whole Live *L. casei* Strains

In experimental models of colon carcinoma, the oral administration of live *L. casei* has been shown to reduce both tumor growth and cellular proliferation. Specifically, daily oral administration of *L. casei* ATCC 393 significantly suppressed tumor volume by 63.1% in BALB/c mice bearing syngeneic subcutaneous CT26 tumors compared to a control group. This reduction was associated with increased cytotoxic activity against cancer cells, as indicated by increased apoptotic markers such as Cleaved Caspase‐3 and poly (ADP‐ribose) polymerase 1 (PARP1) in tumor tissue [21].

Additionally, in a study using an azoxymethane–dextran sodium sulfate (AOM + DSS) model of CRC, oral administration of *L. casei* BL23 significantly protected C57BL/6 mice against cancer development. Macroscopic tumors were absent in the treatment group, while 67% of controls developed tumors. Histological analyses revealed that treated mice had reduced histological scores (*p* = 0.052) and significantly lower Ki67 levels (*p* = 0.044), suggesting decreased proliferation [44]. This antiproliferative effect involved the upregulation of apoptosis‐related genes, including *caspase-7* (*p* = 0.017), *caspase-9* (*p* = 0.028), and *Bik* (*p* = 0.082) [44].

*L. casei* JY300‐8, in combination with *L. reuteri* JMR‐01, significantly inhibited the proliferation of human CRC cell lines (HT29 and HCT116) and murine CT26 cells, with inhibition rates of approximately 72% and 67% at 48 h coculture, respectively. In vivo, oral administration significantly reduced tumor formation rates (by 86.21% with live bacteria and 82.27% with inactivated bacteria) and suppressed tumor growth by ~65%. Importantly, only the live probiotic mixture fully preserved survival at 30 days. Metabolomic profiling linked this suppression to reduced availability of serine, alanine, and nucleotide precursors, thereby constraining pyrimidine and purine metabolism essential for DNA/RNA synthesis [53].

Another study found that a probiotic mix of live *Lactobacillus acidophilus* and *L. casei* sensitized LS513 CRC cells to 5‐fluorouracil‐induced apoptosis in vitro. The presence of 10^8^ CFU/mL of the live bacterial mix increased the apoptotic efficacy of 5‐FU by 40% in a dose‐dependent manner. This combination led to a more rapid activation of Cleaved Caspase‐3 protein [38].

b. *L. casei*–Derived Products and Components

Cell‐free cultural supernatant (CFCS) from *L. casei* has been shown to exert antiproliferative effects. The CFCS from *L. casei* UT1 demonstrated a significant anticancer effect on HCT116 CRC cells in a dose‐ and time‐dependent manner [48]. Microscopic observation revealed cell wrinkling, rupturing, and fragmentation, and fluorescent staining confirmed the incidence of apoptosis, including early and late stages. Flow cytometry further indicated an increase in cells in the sub‐G1 phase from 4.6% to 26.3% after 72 h of treatment, which is consistent with apoptotic cell death [48].

Additionally, CFCS from *L. casei* ATCC 334 inhibited the proliferation of Caco‐2/bbe, SKCO‐1, and SW620 colon cancer cells. Notably, this strain demonstrated the most potent tumor‐suppressive activity among the tested *Lactobacillus* species [40]. Furthermore, ferrichrome induced apoptosis in colon cancer cells via increased expression of Cleaved Caspase‐3 and PARP in a dose‐dependent manner, as well as an elevated number of apoptotic cells. The observed effect was mediated through activation of the JNK‐DDIT3 pathway, as inhibition of JNK reduced the tumor‐suppressive effect. Notably, ferrichrome exhibited minimal growth inhibition on noncancerous intestinal cells [40].

CFCSs derived from conjugated linoleic acids overexpressing *L. casei* (LC‐CLAs) significantly reduced CRC cell viability at all tested concentrations compared to CFCSs from wild‐type *L. casei* (LC‐WT) [47]. Transcriptional analysis showed a marked downregulation of key genes involved in tumor cell growth and proliferation, including *CDK1*, *CDK2*, and *CDK6* (up to a 31.11‐fold decrease), *PLK1* (up to a 21.49‐fold decrease), and *SKP2* (up to a 14.68‐fold decrease). In contrast, the proapoptotic genes *BBC3*, *DDIT3*, and *JUN* were significantly upregulated [47].

Furthermore, antimicrobial peptides m2163 and m2386 from *L. casei* ATCC 334 were found to trigger apoptosis in the human CRC cell line SW480. Flow cytometry showed a dose‐dependent increase in the sub‐G1 population of treated SW480 cells [39]. Notably, the addition of a caspase inhibitor, Z‐VAD‐FMK, partially recovered cells from apoptosis, indicating a caspase‐dependent mechanism. Western blot and qPCR analyses indicated that m2163 induced the expression of Fas and TRAILR1, while m2386 induced Fas, TNFR1, and TRAILR1 death receptors. Both peptides induced the expression of mitochondrion‐associated apoptotic proteins, such as Smac, leading to increased cytoplasmic Smac levels and increased mitochondrial Bax [39].

A metallopeptidase (MPL) gene from *L. casei* ATCC 39392, when endogenously expressed under a colon‐specific promoter in human SW480 CRC cells, resulted in 25% inhibition of proliferation at 48 h [49]. This cytotoxic effect was mediated through apoptosis, with approximately 90% of SW480 cells showing apoptotic cell death by TUNEL staining. Gene expression analysis indicated that MPL expression upregulated *TP53* and *MAP2K1* genes in SW480 cells [49].

In another study, the polysaccharide of *L. casei* SB27 reduced colon cancer in AOM + DSS–induced mice, significantly decreasing tumor number, size, and pathological changes. It also increased Caspase‐3/8/9 activity levels [51]. Additionally, the polysaccharide promoted mitochondrial damage by inducing calcium ion entry, significantly increasing mitochondrial [Ca2+]i and [Ca2+]m in HCT116 cells. This mechanism involved the HINT2/MCU signaling pathway, with the polysaccharide significantly inducing HINT2 mRNA and protein expressions and suppressing HINT2 ubiquitination. Upregulation of HINT2 was shown to reduce cell growth and metastasis, inhibit EdU cell number, and increase Caspase‐3/8/9 activity [51].

Furthermore, selenium nanoparticles (SeNPs) synthesized using *L. casei* (LC‐SeNPs) were discussed in two studies. In one study, using LC‐SeNPs demonstrated anticancer activity on HT29 colon cancer cells [54]. Flow cytometry revealed that LC‐SeNPs induced 23% apoptosis in HT29 cells (10% early and 13% late apoptosis). DAPI staining showed nuclear chromatin condensation, characteristic of apoptosis, in treated cells [54]. Furthermore, LC‐SeNPs treatment led to cell cycle arrest in the sub‐G1 phase and elevated the expression levels of *BAX*, *CASP3*, and *CASP9* genes. It is noteworthy that LC‐SeNPs exhibited minimal toxicity to normal human foreskin fibroblast cells [54].

In another study, SeNPs produced by *L. casei* ATCC 393, as well as SeNP‐enriched *L. casei* (LCSe) and *L. casei*, were found to inhibit murine (CT26) and human (HT29) colon cancer cell growth in a time‐ and concentration‐dependent manner [50]. In vivo, LCSe exhibited the most potent shift from live to apoptotic/dead cell fractions. Notably, SeNPs were nontoxic to primary murine healthy colonic epithelial cells, maintaining a growth rate similar to that of control cells, whereas they inhibited CT26 cancer cell growth by 60%. LCSe also significantly inhibited CT26 cell proliferation more effectively than primary cells, while *L. casei* demonstrated similar antiproliferative effects on both.

SeNPs upregulated proteins involved in redox regulation and apoptosis, including PON2 (threefold increase), catalase (threefold increase), and TRAIL death receptors DR4 and DR5 (1.5‐fold increase). Conversely, SeNPs downregulated HIF‐1*α* (0.5‐fold decrease) and cytochrome c (0.34‐fold decrease) in HT29 cells. Elevated intracellular reactive oxygen species (ROS) levels were also observed in SeNP‐treated HT29 cells [50].

Another *L. casei* strains, particularly LC232, were investigated by Awaisheh et al., which showed strong cytotoxicity against CRC lines Caco‐2 and HRT‐18, with the low half maximal inhibitory concentration (IC_50_) values and significant time‐dependent reductions in cancer cell viability, but no toxicity toward normal cells [41].

Finally, a mucin‐binding protein (MucBP) from *L. casei* CAUH35 inhibited cancer cell proliferation in a variant‐specific manner. The arginine‐containing variant (MucBP36R) reduced HT29 CRC cell growth dose‐dependently, starting at 25 *μ*g/mL, while the serine‐containing variant, MucBP36S, showed no effect up to 200 *μ*g/mL. Control tests with bovine serum albumin confirmed MucBP36R’s specificity [52].

#### 3.3.2. Immunomodulatory Effects of *L. casei* Strains in CRC Models

Various *L. casei* strains and their derivatives demonstrate distinct immunomodulatory effects within CRC models, influencing cytokine profiles, immune cell populations, and pathways associated with tumor progression.

a. *L. casei* ATCC 393 and Derivatives

In an experimental colon carcinoma model in BALB/c mice, oral administration of *L. casei* ATCC 393 led to a significant increase in interferon gamma (IFN‐*γ*) in Peyer’s patches (PPs) 3 and 7 days post–CT26 inoculation. Interleukin‐12 (IL‐12) levels were also elevated in PP on Day 13, although undetectable 7 days post–cancer cell injection. No significant differences were detected in IL‐10 levels in the PP. Analysis of spleen cells revealed a 2.5% increase in CD45+ CD8+ T cells in probiotic‐fed mice compared to controls, with no distinguishable differences in CD4+ T cells or NK cell populations. Restimulation of splenocytes with *L. casei* in vitro showed an increase in IFN‐*γ* secretion, more prominent 7 days post–cancer cell injection, while IL‐10 production was unaffected [21].

Moreover, within the tumor microenvironment, *L. casei* ATCC 393 administration resulted in a significant increase in IFN‐*γ* production. Also, levels of IL‐12p40 were slightly but significantly increased, and a twofold increase in Granzyme B accumulation was observed. Additionally, tumor immunophenotype analysis indicated a significant increase in tumor‐infiltrating lymphocytes, particularly an almost fourfold increase in CD3 + CD8+ cytotoxic T cells. While CD3 + CD4+ helper T cells accumulated, this difference was not statistically significant, and no significant NK cell infiltration was observed. Further analysis showed increased production of proinflammatory interleukins such as IL‐1*β* and IL‐16 and accumulation of various chemotactic agents, including CCL3, CCL4, CCL5, CXCL9, CXCL10, and CXCL11, in the tumor tissue [21].

In another study, preventive administration of *L. casei* 393 improved histopathological features in a 1,2‐dimethylhydrazine (DMH)‐induced BALB/c mouse model of colon cancer, reducing preneoplastic changes and inflammation in distal colon sections. DMH treatment significantly increased serum levels of IFN‐*γ*, TNF‐*α*, IL‐10, IL‐2, IL‐4, and IL‐17A. However, preventive *L. casei* administration significantly decreased IFN‐*γ*, TNF‐*α*, and IL‐10 levels in comparison to the DMH‐only group. IL‐6 levels followed a similar trend but without reaching significance [45].

Interestingly, IL‐2 and IL‐4 levels tended to increase in the *L. casei* preventive group compared to the DMH group, and IL‐17A also tended to increase, with both DMH and *L. casei* preventive groups showing significantly higher levels than healthy controls [45].

b. LC‐WT

CFCSs from LC‐WT and LC‐CLA showed distinct effects. LC‐CLA CFCSs significantly reduced proinflammatory factors (e.g., IL‐1*β*, IL‐6, and TNF‐*α*) and increased anti‐inflammatory cytokines (IL‐10 and TGF‐*β*) in HCT116 cells, outperforming LC‐WT. In mice, LC‐WT downregulated IL‐1*β*, INF‐*γ*, and TNF‐*α* and upregulated IL‐10 and TGF‐*β* in the spleen, while LC‐CLA produced stronger, consistent effects on inflammatory cytokines, with minor variations in IL‐17 and IL‐22 transcription [47].

c. *L. casei* Strain Shirota (*LcS*)

In an AOM‐induced colon cancer model in rats, oral administration of viable *LcS* significantly recovered CD8‐positive lymphocytes to the levels in the control group at 8 and 12 weeks. No significant difference was found in the percentages of CD3‐ and CD4‐positive lymphocytes between the control and *LcS* groups [36].

d. Polysaccharide of *L. casei* SB27

In an AOM + DSS–induced mouse model, a polysaccharide derived from *L. casei* SB27 significantly suppressed the mRNA expressions of IL‐6, IL‐17A, and *ptgs2*. This finding suggests that *L. casei* SB27 polysaccharide exhibits antitumor activity against colon cancer by reducing inflammatory cytokines [51].

#### 3.3.3. Gut Microbiome and Intestinal Barrier Function Modulation

Different strains of *L. casei* have demonstrated varying effects on gut microbiome composition and intestinal barrier integrity, which are linked to CRC progression or prevention in experimental models.

a. Gut Microbiome Modulation

Oral administration of *L. casei* strains has been observed to modulate the gut microbiota composition in mouse models. In AOM + DSS–induced mice, *L. casei* SB27 reduced the abundance of *Fusobacterium nucleatum* and *Bacteroidetes* [51]. Similarly, *L. casei* BL23 treatment in AOM + DSS–induced C57BL/6 mice tended to diverge the microbiota’s beta diversity and tended to restore alpha diversity that was reduced by AOM injection. In this model, *Firmicutes* became the dominant phylum with *Bacteroidetes* ranking second in *L. casei* BL23–treated mice, contrasting with control groups where *Bacteroidetes* was most abundant. *L. casei* BL23 treatment also led to the overrepresentation of *Prevotella*, *Ruminococcaceae*, and *Lactobacillus zeae* [44].

Additionally, LC‐WT and LC‐CLA resulted in significant modulation of gut microbial communities [47]. LC‐CLA specifically boosted the relative abundance of *Firmicutes* and *Thermotogae*, while reducing *Bacteroidetes*, *Proteobacteria*, and *Verrucomicrobia*. At the genus level, LC‐CLA decreased *Bacteroides* and increased *Lactobacillus* and *Bifidobacterium*. LC‐CLA also induced significant shifts in other genera, including higher abundances for *Akkermansia* and *Butyrivibrio*, and lower abundances for *Clostridium* and *Helicobacter*. Notably, LC‐CLA pretreatment decreased the relative abundance of sulfidogenic bacteria by 63.34%, including *Desulfotomaculum* and *Desulfosporosinus* [47].

In a separate study, using *L. casei* DSM 20011 (ATCC 393) in DMH‐induced mice leads to enrichment of *Akkermansia* and *Turicibacter* genera in *L. casei*–treated groups compared to non‐*L. casei*–treated groups (*p* < 0.05) [46].

Furthermore, in the study of Xu et al. Microbiome sequencing revealed that *L. casei* treatment enriched beneficial genera such as *Lactobacillus*, *Coprococcus*, *Roseburia*, and *AF12*, while reducing harmful taxa including *Bacteroides*, *Flexispira*, and *Porphyromonas*. These shifts increased alpha diversity and promoted communities capable of producing antitumor metabolites such as succinate and short‐chain fatty acids, which are known to improve epithelial barrier integrity and suppress carcinogenesis. Notably, *Prevotella*, often associated with inflammation, decreased in the live bacteria group, suggesting a role for *L. casei* in dampening proinflammatory dysbiosis [53].

b. Barrier Function Enhancement

*L. casei* ATCC 393, administered preventively to DMH‐induced BALB/c mice, significantly reduced the number of aberrant crypt foci (ACF). This was associated with maintaining putrescine and spermidine levels similar to healthy controls and decreasing ornithine decarboxylase expression [43]. Additionally, in DMH‐induced male Swiss mice, *L. casei* ATCC 393 treatment reduced ACF by five times and showed reduced inflammation and loss of goblet cells to 20% compared to the DMH group. Mechanistically, this strain was found to increase phosphorylated JNK‐1 and decrease *β*‐catenin and phosphorylated GSK3b expression [46].

#### 3.3.4. Detoxification of Carcinogens

Some strains of *L. casei*, such as the DN‐114001 strain, have shown the capacity to adsorb different colorectal carcinogens, such as IQ (2‐amino‐3‐methylimidazo[4,5‐f]quinoline), MeIQx (2‐amino‐3,8‐dimethylimidazo[4,5‐f]quinoxaline), and PhIP (2‐amino‐1‐methyl‐6 phenylimidazo[4,5‐b]pyridine) in laboratory settings. This ability may contribute to their cancer‐preventive properties by reducing exposure to these harmful compounds [37].

Additionally, Xu et al. reported that both live and inactivated *L. casei* JY300‐8 contribute to chemical detoxification within the intestine through significantly reduced intestinal levels of secondary bile acids (cholic acid, chenodeoxycholate, and deoxycholic acid), which are genotoxic and promote DNA damage. In parallel, levels of sphingosine (a precursor for oncogenic S1P) were decreased and result in lowering tumor‐promoting lipid signaling. Importantly, live *L. casei* cultures also increased production of protective metabolites such as 9‐oxo‐octadecadienoic (9‐OxoODE) acid and succinate, which are associated with anticarcinogenic activity. This dual effect results in removing procarcinogens and enhancing detoxifying metabolites [53].

## 4. Future Perspectives

Evidence to date suggests that *L. casei* could serve as a useful adjunct in CRC therapy. Studies describe multiple anticancer actions: It triggers apoptosis through both death receptor and mitochondrial routes, limits cell division by downregulating *CDK*s and *PLK1*, enhances immunity by raising cytotoxic T‐cell infiltration and IFN‐*γ* release, improves epithelial barrier function, and shifts the gut microbiota toward protective taxa while suppressing carcinogen‐linked species. Potent postbiotics (ferrichrome, antimicrobial peptides, SeNPs, and defined polysaccharides) offer additional benefit, and coadministration with agents such as 5‐fluorouracil appears synergistic.

Probiotic use also carries risks that must be weighed before clinical translation. Although usually tolerated in healthy volunteers, some *Lactobacillus* species have caused bacteremia and endocarditis in immunocompromised patients, catheterized individuals, and those with mucosal injury [55–58]. In severe acute pancreatitis, the PROPATRIA trial reported higher mortality and bowel ischemia after probiotic treatment [59]. Other reports show delayed microbiome recovery after broad‐spectrum antibiotics compared with spontaneous recolonization or fecal transplantation [60], an issue in oncology where antibiotics are common.

Therefore, advancing *L. casei* for CRC requires a safety‐first translational pathway. Future studies should combine assessments of both efficacy and safety: tumor apoptosis and proliferation indices, CD8^+^ infiltration, IFN‐*γ* levels, and microbiome markers such as *Fusobacterium nucleatum* abundance, alongside serial blood cultures, resistome analysis, and metabolic panels in vulnerable patients. It seems that developing good manufacturing practices for live biotherapeutics, combined with the development of drug‐like postbiotics, may provide safe and effective strategies utilizing *L. casei* to mitigate CRC progression and reduce the risk of recurrence.

## 5. Conclusion

This systematic review indicated the multifaceted anti‐CRC properties of *L. casei.* Preclinical evidence demonstrates its ability to induce apoptosis, suppress cell proliferation, modulate the immune system, restore microbiome balance, enhance barrier function, and detoxify carcinogens. Although human data remain limited, the most commonly studied strains (ATCC 334 and ATCC 393) exhibit strong effects both in vitro and in vivo. Standardized dosage and integration into combination regimens with current therapies are all necessary for translation to clinical use.

## Conflicts of Interest

The authors declare no conflicts of interest.

## Author Contributions

M.H.: conceptualization, data flow management, and manuscript editing. M.H.P.: literature search, study selection, and manuscript preparation. A.V.Y.: quality assessment and data extraction. A.A.: manuscript preparation and quality assessment. A.T.: manuscript preparation. M.A.: manuscript preparation and editing and literature search. T.P.: data extraction and data visualization. A.R.: data extraction and quality assessment. H.G.: manuscript preparation, data flow management, and manuscript editing.

## Funding

No funding was received for this manuscript.

## Data Availability

Data sharing is not applicable to this article as no datasets were generated or analyzed during the current study.
